# Evaluation of the postoperative quality of recovery score in radical prostatectomy with erector spinae plane block: a randomized controlled trial

**DOI:** 10.1186/s12871-026-03749-4

**Published:** 2026-03-21

**Authors:** Meryem Onay, Osman Kaya, Ata Özen, Ümit Akkemik, Birgül Yelken, Mehmet Sacit Güleç

**Affiliations:** 1 Department of Anesthesiology and Reanimation, Eskisehir Osmangazi University Faculty of Medicine, Eskisehir, Türkiye; 2 Department of Anesthesiology and Intensive Care, Southend University Hospital, Essex, UK; 3 Department of Urology, Eskişehir Osmangazi University Faculty of Medicine, Eskisehir, Türkiye; 4 Department of Algology, Eskisehir Osmangazi University Faculty of Medicine, Eskisehir, Türkiye

**Keywords:** Quality of Recovery Score, Postoperative Pain Management, Erector Spinae Plane Block, Wound Infiltration, Open Radical Prostatectomy

## Abstract

**Background:**

Radical prostatectomy (RP) is associated with moderate to severe postoperative dynamic pain. In this study, we aimed to evaluate the effect of erector spinae plane block (ESPB) on postoperative Quality of Recovery (QoR), opioid consumption and pain scores in patients who underwent open RP.

**Methods:**

This prospective randomized controlled study included 52 patients who underwent open RP. Patients were randomized into Group E (*n* = 26) with ESPB and Group W (*n* = 26) with wound infiltration. Patient QoR was measured preoperatively and postoperatively using a 40-item QoR-40 questionnaire. Additionally, tramadol consumption, nausea and vomiting, visual analog scale (VAS), sedation, and hemodynamic parameters were evaluated.

**Results:**

The preoperative and postoperative results of the global QoR-40 questionnaire were statistically similar between the groups (*p* = 0.407). Postoperative VAS_Rest_ and VAS_Movement_ were similar between the groups (*p* = 0.313 and *p* = 0.161, respectively). Tramadol consumption was lower in Group E at 2 and 24 h (*p* = 0.035 and *p* = 0.017, respectively). Intraoperative remifentanil consumption was significantly lower in Group E (*p* = 0.005). Complications, such as intraoperative bradycardia, hypotension, and bradycardia-hypotension, were more common in Group E (*p* = 0.001). Postoperatively, complications such as sedation, nausea, and vomiting, and the need for antiemetics were similar between the groups.

**Conclusion:**

ESPB did not improve the quality of postoperative recovery in patients who underwent RP. However, ESPB reduced opioid consumption while postoperative pain scores remained similar.

**Trial Registration:**

ClinicalTrials.gov, NCT05853133, Date of registration: 02/05/2023.

**Supplementary Information:**

The online version contains supplementary material available at 10.1186/s12871-026-03749-4.

## Introduction

Prostate cancer is the most commonly diagnosed cancer in men [1]. Radical prostatectomy (RP) is the primary treatment option recommended to improve survival in patients with localized prostate cancer [2]. Optimal pain management positively affects postoperative recovery, and RP is associated with moderate dynamic pain during the early postoperative period. In recent years, in accordance with the procedure-specific postoperative pain management (PROSPECT) guidelines for open prostatectomies, perioperative opioid-reducing systemic analgesics (paracetamol and nonsteroidal agents) and wound infiltration have been recommended because they are effective, safe and easy to implement [2, 3, 4]. Wound infiltration, particularly in abdominal surgeries, owing to the origin of somatic pain from the deeper layers of the abdominal wall, subcutaneous and subfascial injections are recommended [5].

The Quality of Recovery (QoR) score is a patient-reported outcome measure designed to evaluate postoperative recovery in a multidimensional manner, extends beyond pain assessment alone. It reflects physical comfort, emotional state, psychological support, physical independence, and pain, thereby providing a comprehensive evaluation of early postoperative well-being. The QoR-40 questionnaire, developed by Myles et al. in 2000, is among the most extensively validated tools for this purpose and has been widely used across various surgical populations [6]. Owing to its comprehensive structure and the availability of a validated Turkish version, the QoR-40 was preferred over shorter recovery assessment tools in the present study, particularly given the complexity and recovery burden associated with open RP [7].

Erector spinae plane block (ESPB), a frequently used facial plane block, was first described by Forero for neuropathic pain. The block is achieved by applying local anesthetic into the interfascial space between the erector spinae muscle and the thoracic transverse process. It is hypothesized that in the block, the local anesthetic spreads into the epidural or paravertebral space, resulting in the blockade of the dorsal and ventral branches of the thoracic and abdominal nerves as well as the sympathetic ganglia. ESPB has been increasingly applied in thoracic and abdominal surgeries and has been shown to reduce postoperative pain and opioid consumption [5, 8, 9, 10].

Despite the growing body of evidence supporting the analgesic efficacy of ESPB, data regarding its effect on postoperative QoR, particularly in patients undergoing open RP, remain limited. Moreover, wound infiltration continues to be recommended as a standard analgesic technique for this procedure. On the basis of the potential of ESPB to attenuate both somatic and visceral pain, we hypothesized that, compared with wound infiltraion, ESPB would provide superior postoperative analgesia and improve the QoR in patients undergoing open RP.

The primary objective of this study was to compare the effect of ESPB and with wound infiltration on postoperative QoR, which was assessed using the QoR-40 questionnaire, in patients who underwent open RP. Secondary outcomes included postoperative pain scores, opioid consumption, nausea, and vomiting.

## Method

This prospective randomized controlled clinical trial enrolled 52 patients aged 18–70 years, who were classified according to the American Society of Anesthesiologists (ASA) physical status I-III, and wjo underwent open RP at Eskisehir Osmangazi University Hospital, Turkey between May 2023 and February 2024. The trial was approved by the Clinical Research Ethics Committee (ID: 2023/42). The study was conducted in accordance with the Helsinki Declaration and the CONSORT guidelines for reporting randomized trials. The study was registered at ClinicalTrials.gov (ID: NCT05853133). Written and verbal informed consent was obtained from all patients. The exclusion criteria were infection at the incision site, coagulation disorders, a history of allergy to the drugs used in the study, insufficient cognitive capacity to adequately assess pain using the visual analog scale (VAS), the use of patient-controlled analgesia, and a body mass index (BMI) ≥ 35 kg/m².

### Randomization and blinding

Postoperative analgesia techniques were divided into two groups: ESPB (Group E) and wound infiltration (Group W). A total of 53 patients were randomized in the study. One patient in the ESPB group was excluded before analysis because of block failure, resulting in exclusion before the primary outcome assessment. Consequently, 52 patients were included in the final analysis for the primary outcome, with 26 patients analyzed in each group (Group E: ESPB, *n* = 26; Group W: wound infiltration, *n* = 26).

Randomization was performed using a computer-generated random number sequence generated at Randomization.org (https://www.randomization.org), and group allocation was concealed using sealed, opaque envelopes. The investigators responsible for postoperative follow-up, data collection, and administration of the QoR-40 questionnaire were also blinded to group allocation (O.K). Owing to the nature of the interventions, blinding of the anesthesiologist performing the ESPB and the surgeon was not feasible. However, these clinicians were not involved in postoperative outcome assessment or data analysis.

### General anesthesia procedure

Before the operation, the patients were informed of the visual pain scale and patient-controlled analgesia. A standard general anesthesia protocol was implemented after routine electrocardiography, peripheral oxygen saturation, noninvasive blood pressure, and bispectral index (BIS) monitoring. After preoxygenation, 2–3 mg/kg propofol, 0.8 mg/kg rocuronium and 1 mcg/kg fentanyl were administered for anesthesia induction. Considering the average age of the study population, the propofol dosage was individualized and titrated according to clinical response and BIS monitoring, and lower doses were administered when appropriate. For anesthesia maintenance, sevoflurane, O_2_/air (FiO_2_:0.40), and a 0.1–0.25 mcg/kg/min remifentanil infusion were used. Heart rate and blood pressure were titrated by adjusting the remifentanil infusion to maintain values within 20% of baseline. Ephedrine (5 mg) was administered when the patient’s systolic blood pressure decreased by more than 30% from baseline or when the mean arterial pressure fell below 65 mmHg. Atropine (0.5 mg) was administered if the heart rate decreased below 50 beats per minute. The BIS score was maintained between 40 and 50. All patients were administered an intravenous infusion of a balanced solution (5–7 mL/kg). The autoflow volume-controlled mode (Dräger Perseus^®^ A500e) was preferred for mechanical ventilation. The respiratory rate was adjusted to achieve a tidal volume of 7–8 mL/kg, an I: E ratio of 1:2, and an end-tidal carbon dioxide (EtCO_2_) concentration ranging from 30 to 40 mmHg. To provide easy access to the surgical site, the patients were positioned to achieve lumbar extension. Open retropubic RP was performed all patients by the same surgical method and by the same person (A.Ö). The procedure comprised midline infraumbilical incision, opening of the endopelvic fascia, control of the dorsal venous complex, nerve-sparing dissection, apical urethral dissection, ligation of the lateral pedicles with excision of the seminal vesicles, eversion of the bladder mucosa followed by urethrovesical anastomosis, bilateral iliac and obturator lymph node dissection, separation of the prostate from the bladder neck, and complete removal of the prostate. Thirty minutes before the surgery was completed, all patients were administered 1 g paracetamol and 1 mg/kg tramadol. At the end of surgery, 2 mg/kg sugammadex was administered to reverse the neuromuscular blockade. Patients admitted to the postanesthesia care unit (PACU) after extubation were discharged from the PACU when their Modified Aldrete Score was ≥ 9.

### Erector spinae plane block

**Fig. 1:**
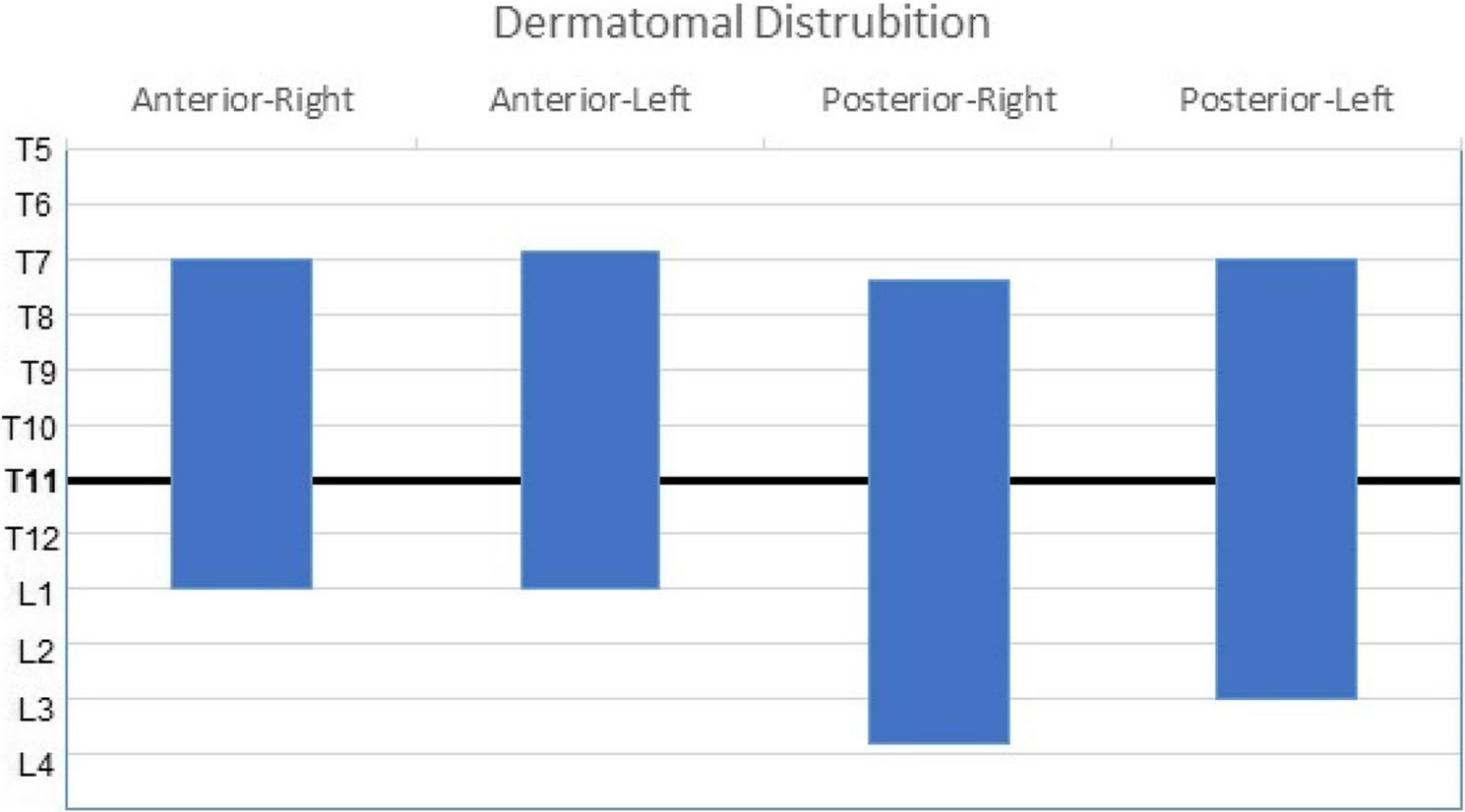
Dermatomal distribution of the sensory block after erector spinae plane block

Patients scheduled for ESPB were brought to the regional anesthesia room 45 min before surgery. ESPB was administered by the same anesthesiologist (M.O) in the prone position under sedation (0.03 mg/kg midazolam and 50 mcg fentanyl), following standard ASA monitoring. Ultrasonography was performed using a 2–4 MHz convex probe (Philips Affiniti 50, Philips Medical Systems, Seattle, WA, United States) at the T11 level, scanning laterally 3 cm from the midline. The transverse process and erector spinae muscles were also identified. A 21-gauge, 85-mm needle (Vygon (UK) Ltd, Swindon, UK) was inserted using an in-plane technique in the cranio-caudal direction. After the needle made contact with the transverse process, anatomical localization was confirmed using 1–2 mL of saline for hydrodissection. A total of 40 mL 0.25% bupivacaine, with 20 mL 0.25% bupivacaine administered on each side, was applied bilaterally to the fascial plane on the deep surface of the erector spinae muscle. Dermatomal spread was assessed 30 min after the block using pimprick and cold-hot testing along both the anterior (midclavicular) and posterior lines. A successful block was defined as the achievement of at least four dermatomal involvements that included the retropubic incision (T10-L1), as shown in Fig. 1.

### Wound infiltration

At the end of surgery, the surgeon performed wound infiltration under general anesthesia. For this, 20 mL 0.25% bupivacaine was administered subcutaneously, and 20 mL 0.25% bupivacaine was administered subfascially. To avoid intravascular injection, the dose was administered multiple times using a volume of 5 mL each time with negative blood aspirations after each administration.

### Outcome measurement

The primary outcome of this study was the quality of postoperative recovery, which was assessed using the 40-item Qulaity of Recovery (QoR-40) questionnaire administered 24 h after surgery.

The QoR-40 questionnaire, developed by Myles et al. [6], is a validated patient-reported outcome measure designed to assess early postoperative recovery across five domains: emotional state (9 items), physical comfort (12 items), psychological support (7 items), physical independence (5 items), and pain (7 items). Each item is scored on a 5-point Likert scale, yielding a total score ranging from 40 (poor recovery) to 200 (excellent recovery). Patients completed the questionnaire preoperatively in the waiting area and again at 24 h postoperatively. The Turkish version of the QoR-40 has been validated by Karaman et al. and was used in this study [7].

The secondary outcomes included postoperative pain scores at rest and during movement; total opioid consumption; intraoperative remifentanil consumption; hemodynamic parameters (heart rate, systolic, diastolic and mean blood pressure and peripheral oxygen saturation); occurence of nausea and vomiting; sedation scores; and postoperative complications. Pain intensity was assessed using the VAS (0–10). All patients received intravenous patient-controlled analgesia with tramadol (bolus dose 10 mg, no basal infusion) and paracetamol 1 g every 8 h. Rescue analgesia with 2 mg intravenous morphine was administered when the VAS score ≥ 4. Nausea and vomiting were evaluated using a 4-point scale, and 8 mg ondansetron was given when the score was ≥ 2. All secondary outcomes were recorded at 0, 2, 6, 12, and 24 h postoperatively.

### Sample size

A priori power analysis was conducted using G*Power 3.1 software to determine the required sample size for detecting a statistically significant difference between two independent groups. On the basis of data obtained from an initial pilot study with seven participants per group, the mean and standard deviation values of the 40-item QoR-40 questionnaire were as follows: Group E: mean = 185.9, SD = 3.6; Group W: mean = 179.6, SD = 8.7. The effect size (Cohen’s d) was calculated as 0.95. The power analysis was performed for a two-tailed analysis with an alpha level of 0.05 and a desired power of 0.90. The results indicated that a total sample size of 50 participants (25 per group) is needed to achieve adequate statistical power.

### Statistical analysis

The normality of the distribution of continuous variables was evaluated using the Shapiro–Wilk normality test. For normally distributed variables summarized by mean +- standart deviation, nonnormaly distributed variables summarized by median and Inter Quartile Range (IQR). For normally distributed variables were compared between groups by independent samples t test and nonmormaly distributed variables were compared by the Mann–Whitney-U test. Categorical variables between groups were compared using Yates, Fisher’s exact tests and Monte Carlo exact chi-square analyses. The level of significance was set at *p* < 0.05. Statistical analyses were performed using IBM SPSS Statistics Version 27.0 (IBM Corp., Armonk, NY, USA).

## Results

**Fig. 2:**
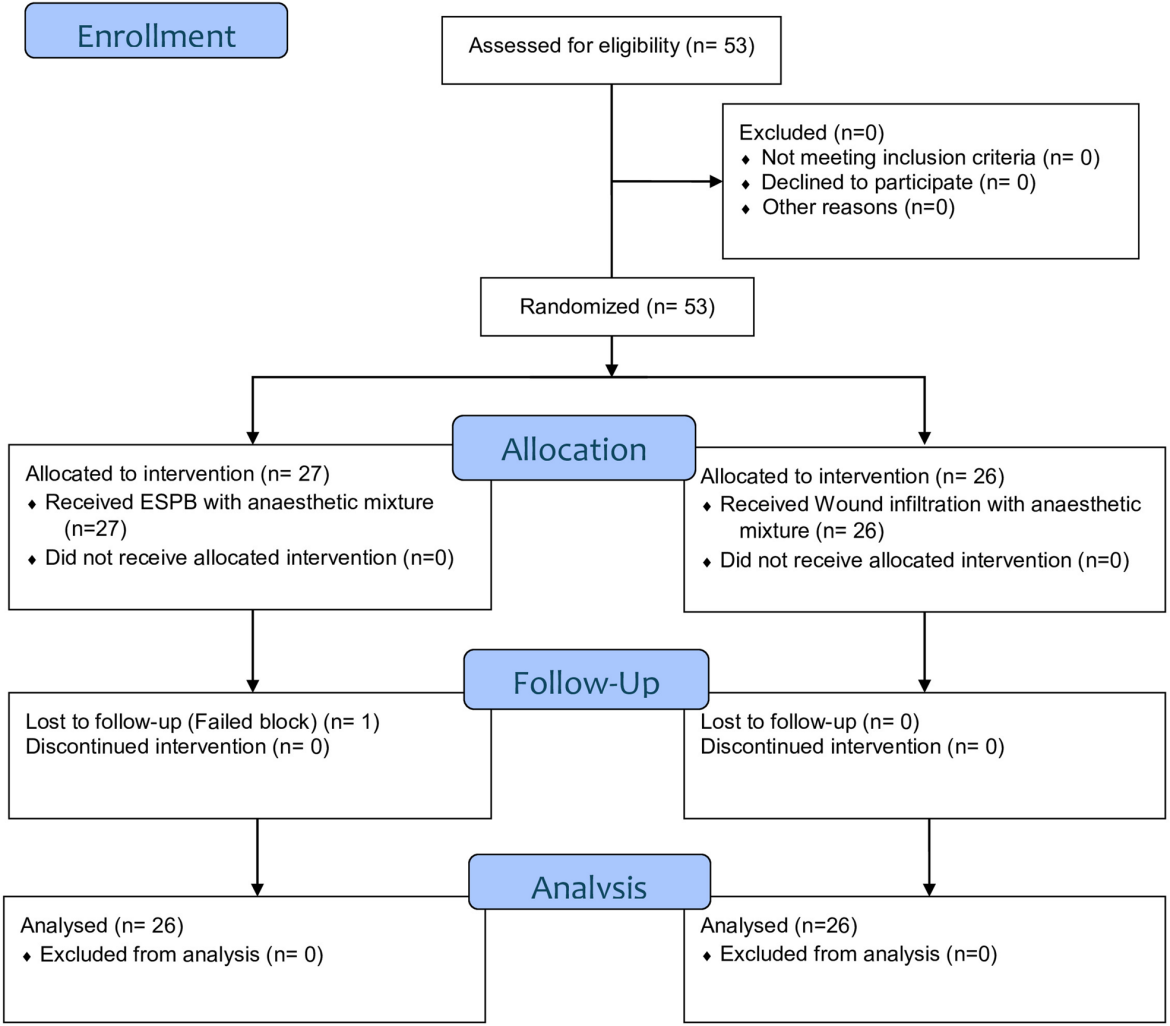
CONSORT flow diagram

In this study, patients who did not achieve the intended dermatomal spread following ESPB administration were excluded. Sensory mapping performed 30 min after ESPB demonstrated consistent involvement of the T10–L1 dermatomes, corresponding to the surgical incision area (Fig. 1). In total of 52 patients were randomized (Group E, *n* = 26; Group W, *n* = 26). This information is presented in the CONSORT diagram (Fig. 2). Patient demographic data such as age, body mass index (BMI), ASA score, operation duration, intraoperative remifentanil consumption, and undesirable effects such as bradycardia-hypotension are presented in Table 1.

**Table 1:** Baseline, demographic and surgical characteristics

	Group E (*n*:26)	Group W (*n*:26)		Difference in means, 95%CI
AgeBMIASA I/II/III	63.04 ± 5.04 26.54 ± 2.38 5/18/3	62.46 ± 6.51 25.81 ± 3.53 2/23/1	0.722* 0.386* 0.264**	-0.577 (-3.821 -2.667) -0.731 (-2.410-0.949)
Operative time Remifentanil Consumption (mL)	203.35 ± 21.30 25.08 ± 10.85	214.81 ± 33.30 41.54 ± 23.50	0.146* 0.002*	11.462 (-4.111-27.034) 16.462 (6.263–26.660)
Bradycardia-Hypotension Bradycardia Hypotension	6 (%23.1) 3 (%11.5) 1 (%3.8)	0 (%0) 3 (%11.5) 5 (19.2)	0.023**	
Rescue analgesia (24 h) Antiemetic requirement	5 (%19.2) 4 (%15.4)	10 (%38.5) 7 (%26.9)	0.221**** 0.499***	

Data are expressed as mean ± SD or number (%)

*BMI* Body mass index, *ASA* American Society of Anesthesiologists

*Independent samples t test, **Chi-square Monte Carlo exact test, ***Fisher’s Exact test, ****Yates’ test

The primary outcome and the QoR-40 questionnaire results, including the total scores and individual components, are presented in detail in Table 2. The global QoR-40 questionnaire results were not significantly different between the groups during either the preoperative or postoperative period (*p* = 0.407). Parameters such as emotional state, comfort, physical independence, and pain were not significantly different. As shown in Table 2, the psychological support domain of the QoR-40 questionnaire was significantly different between the groups (*p* < 0.05).

**Table 2:** Total and dimensional QoR-40 scores

QoR40	Group		*P* value*
	Group E (*n*:26)	Group W (*n*:26) Median (IQR)	
PREOPERATIVE			
Physical comfort	55 (2.75)	59 (3.75)	0.526
Emotional status	44 (4)	44 (3)	0.604
Physical independence	25 (0)	25 (0)	0.600
Psychological support	35 (1)	35 (0)	0.002
Pain	35 (1)	35 (1)	1.000
Global QoR-40	196.50 (8.5)	198 (7)	0.428
POSTOPERATIVE			
Physical comfort	54 (6.75)	54 (4.75)	0.686
Emotional status	43 (3.75)	43 (3.50)	0.752
Physical independence	23 (3.75)	21.5 (4.5)	0.216
Psychological support	35 (1)	35 (0.75)	0.588
Pain	32 (2.75)	32.5 (3.75)	0.889
Global QoR-40	186.5 (10)	187 (13.75)	0.949

Data are expressed as median (IQR)

*Mann- Whitney U test

The VAS_Rest_ and VAS_Movement_ scores at 24 h postoperatively were similar between the groups (Table 3). No statistically significant differences were observed at any of the time points.

**Table 3:** Assessment of the VAS scores of the groups at rest and during movement

Time	Group	*P* value*	
	Group E (*n*:26)	Group W (*n*:26)	
VAS_Rest_ 2 h 6 h 12 h 24 h	4 (0.75) 2.5 (2) 2 (2) 2 (1.75)	4 (1) 3 (2) 3 (1) 2 (1.75)	0.415 0.693 0.123 0.163
VAS_Movement_ 2 h 6 h 12 h 24 h	5 (2) 4.5 (3,75) 3 (1) 4 (2.75)	5 (1) 5 (2) 4 (2) 4 (3)	0.219 0.963 0.216 0.527

Visual analog scale scores of the groups at rest and during movement are expressed as the median (IQR)

*Mann- Whitney U test

Tramadol consumption was compared between the groups and is presented in Table 4. Tramadol consumption was significantly lower in Group E at 2 and 24 h (*p* = 0.035 and *p* = 0.017, respectively). The need for rescue analgesia during the first two postoperative hours was 19.2% in Group E and 38.5% in Group W, but there was no significant difference between the groups (*p* = 0.221) (Table 1). Intraoperative remifentanil consumption was lower in Group E (*p* = 0.005). Intraoperative bradycardia, hypotension, and bradycardia-hypotension were significantly more frequent in Group E (*p* = 0.001). There were no significant differences between the groups in terms of complications, such as sedation, nausea, vomiting, or antiemetic requirements, during the postoperative period (Table 1).

**Table 4:** Assessment of postoperative tramadol consumption

Time (hour)	Group W (*n*:26)	Group E (*n*:26)	*p* value	Difference in means, 95%CI
2 h	4.12 ± 1.33	2.96 ± 2.35	0.035*	1.154 (0.086–2.221)
6 h	8.54 ± 3.82	6.23 ± 4.99	0.068	2.308 (-0.172-4.788)
12 h	12.96 ± 7.36	10.69 ± 8.20	0.299	2.269 (-2,072-6.610)
24 h	21.88 ± 11.29	14.58 ± 9.94	0.017*	7.308 (1.380-13.235)

Data are presented as mean ± SD

*p* ≤ 0.05 was considered to indicate statistical significance

## Discussion

Regional analgesic techniques have been increasingly investigated for their potential to improve postoperative recovery and reduce opioid consumption after major abdominal surgery [2, 3]. In this study, the effects of ultrasound-guided ESPB and wound infiltration as post-operative analgesic techniques on postoperative recovery quality in patients who underwent open RP were evaluted. ESPB did not improve the quality of postoperative recovery compared with wound infiltration in patients who underwent RP. In patients who underwent ESPB, the total opioid consumption durring the 24-h postoperative period was lower than that in patients who underwent wound infiltarion. Postoperative pain scores and the incidence of nausea and vomiting were similar between the groups.

ESPB has recently become popular among fascial plane blocks owing to its clinical efficacy and safety [8, 9]. Although various hypotheses regarding its mechanism of action are still debated, the analgesic effect is attributed mainly to the spread of local anesthetic within the deep fascial plane of the erector spinae muscle, with possible extension to the epidural and paravertebral spaces and systemic effects [2, 9]. The erector spinae muscle extends along the thoracolumbar spine, allowing for wide craniocaudal spread and the involvement of multiple dermatomes, which supports its use in both acute and chronic pain conditions [9, 11]. However, the spread of local anesthetic may be influenced by technical factors such as the level of injection and anatomical characteristics, particularly the increasing thickness of the muscle at lower thoracic levels, which may affect hydrodissection and drug distribution [2, 9, 12]. Therefore, in our study, ESPB was administered preoperatively and block success was confirmed by dermatomal assessment, with involvement of at least four dermatomes covering the surgical incision (T10–L1).

Wound infiltration blocks the somatic afferent fibers of the abdominal wall, thereby providing pain control [13]. Owing to a decrease in opioid use, early mobilization, increased bowel motility, and lower complication risk, multimodal analgesia has recently been implemented as a component of postoperative pain control following surgical procedures [6]. In this study, wound site infiltration was preferred over ESPB because of its routine recommendation as a technique for open RP and its ability to prevent somatic pain [3].

Open RP can cause both visceral and somatic pain [2, 3]. The incision extending from below the umbilicus to the pubis (T10–L1) and manipulation of muscle and fascial tissues contribute to somatic pain, whereas visceral pain originates from the hypogastric and pelvic plexuses that provide sensory innervation to the prostate. Dost et al. emphasized that effective relief of visceral pain may require blockade of the sacral roots and reported that ESPB administered at the T11 level was insufficient for postoperative analgesia in RP [2]. In contrast, other prostatectomy-specific studies have suggested that ESPB administered at lower thoracic levels may still provide clinically relevant analgesia.

However, in the the sympathetic system, the sympathetic chain of the spinal cord connects through 14 spinal nerves exiting the intervertebral foramina between T1 and L3 via the T1-L2 roots. The sympathetic supply to the abdomen is provided by the ganglion (such as the hypogastric and impar) formed by branches originating from T7-11 [14]. Therefore, ESPB performed at the T11 level may contribute to visceral analgesia through a possible sympathetic mechanism, although this remains unclear and was not directly evaluated in the present study.

Tulgar et al. demonstrated that ESPB administered at the level of the lower thoracic spine (T12) under ultrasound guidance led to effective and long-lasting postoperative analgesia in patients who underwent radical retropubic prostatectomy [15]. Although the number of randomized controlled trials evaluating ESPB for postoperative analgesia after open RP remains limited, Turan et al. reported that ESPB applied at the T11 level as part of multimodal analgesia reduced intraoperative and postoperative analgesic consumption and pain scores, indicating a beneficial effect on pain control in patientswho underwent open RP [16]. In the study conducted by Składzień et al., both the ESPB performed at the T9 level and the quadratus lumborum block were associated with effective postoperative analgesia in patients undergoing laparoscopic prostatectomy. No statistically significant differences were observed between the two interfascial plane blocks in terms of opioid consumption or pain scores [17]. Consistent with the results of these prostatectomy-specific studies, total opioid consumption at 2 and 24 h postoperatively was significantly lower in Group E compared with wound infiltration in our study. This finding suggests that ESPB provides extended analgesic efficacy for up to 24 h after open RP. Moreover, the reduction in opioid requirements, together with the occurrence of intraoperative bradycardia–hypotension, supports the notion that ESPB may be associated with attenuation of both somatic and visceral pain components, although the underlying mechanisms were not directly evaluated. Therefore, ESPB administered at the T11 level may be considered an effective analgesic technique for reducing postoperative opioid consumption following open RP.

ESPB has been shown to provide sufficient analgesia in thoracic, breast, bariatric, and upper abdominal surgeries, depending on the level of application [18, 19, 20]. Although there have been significant improvements in postoperative analgesia and a reduction in opioid consumption, their impact on the QoR is still debated. Delayed return to normal activities, along with patient dissatisfaction, can lead to prolonged recovery and delayed discharge, which, in turn, increases costs. The 40-item QoR-40 questionnaire is an accepted tool for assessing QoR in many surgical procedures [19, 21].

In this study, it was hypothesized that, considering the surgical position of the patients in major surgeries such as open RP, there may be an impact on patient comfort, potential exacerbation of joint complaints with lumbar extension, and a negative effect on the quality of patient recovery. A cadaveric study demonstrated that the ESPB extended medially to the facet joints and laterally to the thoracolumbar fascia, in addition to its craniocaudal spread [22]. The Lumbar ESPB has been found to be effective for treating chronic low back pain accompanied by postdisc surgery or radicular symptoms [23, 24].

The parameters in the QoR survey, in addition to psychological support, were dependent on the patient’s clinical and emotional status. Although ESPB reduced intraoperative opioid and postoperative tramadol consumption, this analgesic benefit alone may not be sufficient to influence overall postoperative recovery. In our study, although there was a tendency toward a decrease in emotional status, physical comfort, pain, and physical independence scores on the QoR-40 questionnaire after surgery and anesthesia, no statistically significant differences were observed between the groups. Overall, compared with wound infiltration, ESPB did not result in superior quality of postoperative recovery as reflected by similar global QoR-40 scores between the groups. Although ESPB reduced intraoperative opioid requirements and postoperative tramadol consumption, this opioid-sparing effect did not translate into an improvement in overall recovery, likely because QoR is a multidimensional outcome influenced by factors beyond pain control alone and because recovery was assessed only during the early postoperative period (first 24 h). Accordingly, the statistically significant reduction in tramadol consumption appears to be clinically relevant mainly as an opioid-sparing effect rather than an improvement in overall recovery. Future large-scale, multicenter randomized trials are warranted to confirm these opioid-sparing benefits and to better define the role of ESPB in postoperative analgesia after open radical prostatectomy.

This study has several limitations. First, the study was conducted at a single center. Second, complete blinding was not feasible because ESPB was performed preoperatively to assess block success using dermatomal sensory examination, which may have introduced potential bias. Although the use of a placebo group has been suggested in previous studies to reduce bias, this approach was considered potentially unsafe for patients. Therefore, instead of a placebo, wound site infiltration, which is a simple and guideline-recommended technique for postoperative analgesia, was used as the control intervention. In addition, the ESPB was performed preoperatively, whereas wound infiltration was applied at the end of surgery; this difference in timing may have influenced analgesic outcomes and hemodynamic changes.

QoR was assessed only within the first 24 h postoperatively using the QoR-40 questionnaire; therefore, longer-term recovery outcomes could not be evaluated. Finally, the sample size calculation was based on the primary outcome (quality of recovery), and no separate power analysis was performed for secondary outcomes. As a result, the study may have been underpowered to detect differences in secondary endpoints, and these results should be interpreted with caution.

## Conclusion

Compared with wound infiltration, ESPB did not improve the quality of postoperative recovery in patients who underwent RP. ESPB reduced opioid consumption but postoperative pain scores remained similar. Since ESPB reduces opioid consumption, it may be preferred as a component of multimodal analgesia.

## Supplementary Information

Supplementary Material 1.

## Data Availability

Data are available by contacting the corresponding author through email.

## Abbreviations

ASA: American Society of Anesthesiologists
BMI: Body Mass Index
VAS: Visual Analog Scale
RP: Radical Prostatectomy
Global QoR-40: Quality of Recovery Questionnaire
ESPB: Erector Spinae Plane Block

## References

[1] Abdelrazek AS, Ghoniem K, Ahmed ME, Joshi V, Mahmoud AM, Saeed N, et al. Prostate Cancer: Advances in Genetic Testing and Clinical Implications. Uro. 2023;3(2):91–103.

[2] Dost B, Kaya C, Ozdemir E, Ustun YB, Koksal E, Bilgin S, et al. Ultrasound-guided erector spinae plane block for postoperative analgesia in patients undergoing open radical prostatectomy: A randomized, placebo-controlled trial. J Clin Anesth. 2021;72:111–7. 10.1016/j.jclinane.2021.110277 33838536

[3] Lemoine A, Witdouck A, Beloeil H, Bonnet F, Albrecht E, et al. PROSPECT guidelines update for evidence-based pain management after prostatectomy for cancer. Anaesth Crit Care Pain Med. 2021;40:100922. 34197976 10.1016/j.accpm.2021.100922

[4] Elkassabany N, Ahmed M, Malkowicz SB, Heitjan DF, Isserman JA, Ochroch EA. Comparison between the analgesic efficacy of transversus abdominis plane (TAP) block and placebo in open retropubic radical prostatectomy: A prospective, randomized, double-blinded study. J Clin Anesth. 2013;25:459–65. 23965191 10.1016/j.jclinane.2013.04.009

[5] Stamenkovic DM, Bezmarevic M, Bojic S, Unic-stojanovic D, Stojkovic D, Slavkovic DZ, et al. Updates on wound infiltration use for postoperative pain management: A narrative review. J Clin Med. 2021;10:4659. 34682777 10.3390/jcm10204659 PMC8537195

[6] Myles PS, Weitkamp B, Jones K, Melick J, Hensen S. Validity and reliability of a postoperative quality of recovery score: The QoR-40. Br J Anaesth. 2000;84:11–5. 10740540 10.1093/oxfordjournals.bja.a013366

[7] Karaman S, Arici S, Dogru S, Karaman T, Tapar H, Kaya Z, et al. Validation of the Turkish version of the quality of recovery-40 questionnaire. Health Qual Life Outcomes. 2014;12:1–6. 10.1186/1477-7525-12-8 PMC3896711 24428925

[8] Forero M, Adhikary SD, Lopez H, Tsui C, Chin KJ. The erector spinae plane block a novel analgesic technique in thoracic neuropathic pain. Reg Anesth Pain Med. 2016;41:621–7. 27501016 10.1097/AAP.0000000000000451

[9] Chin KJ, El-Boghdadly K. Mechanisms of action of the erector spinae plane (ESP) block: a narrative review. Can J Anaesth. 2021;68:387–408. 33403545 10.1007/s12630-020-01875-2

[10] Moharam SA, ElSharkawy MS, Almohasseb MA, Hamama K, Mahmoud MA, Abogabal MA. Ultrasound-guided external oblique intercostal plane block versus thoracic erector spinae block for post-thoracotomy pain: a randomized double-blinded non-inferior clinical study. Indian J Anaesth. 2025;69:809–15. 40800700 10.4103/ija.ija_3_25 PMC12338484

[11] Cadavid A, Barrios A, Camelo J, Gómez J, Forero M, Peng PWH, et al. Prospective Evaluation of Sensory Mapping of Erector Spinae Plane Block. Pain Physician. 2020;23:E289–298. 32517405

[12] Hong HJ, Park JH, Shim JW. Comparison of Craniocaudal Spread of Lumbar Erector Spinae Plane Block With Two Volumes of Local Anesthetics. IRB. 2022;1:1–7. 37192229

[13] Osaheni O, Idehen HO, Imarengiaye CO. Analgesia for postoperative myomectomy pain: A comparison of ultrasound-guided transversus abdominis plane block and wound infiltration. Niger J Clin Pract. 2020;23:1523–9. 33221776 10.4103/njcp.njcp_162_19

[14] Gifford L, Thacker M. A clinical overview of the autonomic nervous system, the supply to the gut and mind–body pathways. Topical Issues Pain. 2013;3:23.

[15] Tulgar S, Senturk O. Ultrasound guided low thoracic erector spinae plane block for postoperative analgesia in radical retropubic prostatectomy, a new indication. J Clin Anesth. 2018;47:4–9. 29518667 10.1016/j.jclinane.2018.02.013

[16] Turan D, Ozden MGN, Kocoglu H. Effects of ultrasound-guided erector spinae plane block in radical prostatectomy surgery on pain and surgical stress response. Ain-Shams J Anesthesiol. 2023;15:52.

[17] Składzień T, Maciejewski P, Szpunar W, Lonc T, Szpor J, Cicio M, et al. Effects of erector spinae plane block and quadratus lumborum block on postoperative opioid consumption in laparoscopic prostatectomy: a randomized controlled clinical trial. Anaesthesiol Intensive Ther. 2025;57:e267. 10.5114/ait/210670 PMC12793978 41099275

[18] Leong RW, Tan ESJ, Wong SN, Tan KH, Liu CW. Efficacy of erector spinae plane block for analgesia in breast surgery: a systematic review and meta-analysis. Anaesthesia. 2021;76:404–13. 32609389 10.1111/anae.15164

[19] Canıtez A, Kozanhan B, Aksoy N, Yildiz M, Tutar MS. Effect of erector spinae plane block on the postoperative quality of recovery after laparoscopic cholecystectomy: a prospective double-blind study. Br J Anaesth. 2021;127:629–35. 34340839 10.1016/j.bja.2021.06.030

[20] Feray S, Lubach J, Joshi GP, Bonnet F, Van de Velde M, Joshi GP, et al. PROSPECT guidelines for video-assisted thoracoscopic surgery4: a systematic review and procedure‐specific postoperative pain management recommendations. Anaesthesia. 2022;77:311–25. 34739134 10.1111/anae.15609 PMC9297998

[21] Gornall BF, Myles PS, Smith CL, Burke JA, Leslie K, Pereira MJ, et al. Measurement of quality of recovery using the QoR-40: A quantitative systematic review. Br J Anaesth. 2013;111:161–9. 23471753 10.1093/bja/aet014

[22] De Lara González SJ, Pomés J, Prats-Galino A, Gracia J, Martínez-Camacho A, Sala-Blanch X. Anatomical study of the distribution of the volume administered after deep-plane lumbar erector spinae block. Rev Esp Anestesiol Reanim. 2019;66:409–16. 10.1016/j.redar.2019.07.001 31488244

[23] Durmus IE, Surucu S, Muz A, Takmaz SA. The effectiveness of erector spinae plane block in patients with chronic low back pain. Euro Rev Med Pharmacol Sci. 2023;27:138–43. 10.26355/eurrev_202301_30864 36647861

[24] Akyuz ME, Firidin MN. Bilateral ultrasound-guided erector spinae plane block for postoperative persistent low back pain in lumbar disc surgery. Eur Spine J. 2022;3:1873–8. 10.1007/s00586-022-07212-z 35420380

